# Common medium versus advanced IVF medium for cryopreserved oocytes in heterologous cycles

**DOI:** 10.1038/s41420-017-0025-2

**Published:** 2018-02-20

**Authors:** R. Poverini, R. Lisi, F. Lisi, V. Berlinghieri, W. Bielli, P. Carfagna, A. Costantino, D. Iacomino, G. Nicodemo

**Affiliations:** IVF Unit, Casa di Cura Villa Mafalda, Rome, Italy

## Abstract

Granulocyte-macrophage colony-stimulation factor plays different crucial roles during embryo implantation and subsequent development. Here we aimed to evaluate the effects of embryo cell culture medium, with the inclusion of granulocyte-macrophage colony-stimulation factor (GM-CSF), on embryo development and pregnancy rate. To this end, we took advantage of our retrospective observational study to correlate the outcomes from two different culture media. We included in this study 25 unselected patient from our IVF Center that underwent heterologous IVF cycle with crypreserved oocytes. We analyze the fertilization rate, pregnancy rate, and embryo quality at different day of transfer obtained from two different media composition. Our results show that the rate of fertilization and the pregnancy rate were increased using medium added with this particular type of cytokines (GM-CSF).

## Introduction

Culture conditions are crucial for the final outcome in assisted reproductive technologies and they have an impact on pre- and post-implantation development, potentially regulating the future health of the offspring. To provide an optimal environment for zygote/embryos, clinical embryologist has long been involved in optimizing the culture conditions. Previous work^1–3^ has evaluated embryo metabolism, as well as its homeostasis, including focusing on the role of in vitro specific media components in order to determine what, and when, should be added in embryo culture medium. For example that pyruvate, as a powerful antioxidant, is able to reduce intracellular levels of hydrogen peroxide in the embryo^4^. The presence of pyruvate in embryo culture medium confers a significant degree of protection against oxidative stress, as well as serving as a vital energy source. In addition, the concentration of glucose in the culture medium affects its rate of consumption by the embryo^5^; therefore increasing the concentration of glucose in the medium can result in increased glucose uptake and utilization. Devreker et al.^6^ have shown that human embryo development in culture is enhanced when embryos are first exposed to non-essential amino acids followed by a more complex array of amino acids. After the eight-cell stage, embryo benefits from the presence of a more complex array of amino acid being found to stimulate the development of the ICM (inner cell mass)^7^.

All of these components are currently included in several different media used for embryo culture, but the presence of the GM-CSF cytokine in the advanced IVF media has not been fully established so far. The reproductive tissues undergo profound structural changes and major immune adaptation to accommodate pregnancy. Granulocyte-macrophage colony-stimulating factor (GM-CSF) is a crucial cytokine with pivotal roles during embryo implantation and subsequent development^8^. Several cell lineages in the reproductive tract and gestational tissues synthesis GM-CSF under the control of ovarian steroid hormones and in response to signaling agents originating in male seminal fluid and the conceptus. Indeed, the pre-implantation embryo, invading placental trophoblast cells and the abundant populations of leukocytes controlling maternal immune tolerance are all subject to GM-CSF regulation. GM-CSF deficiency in pregnancy adversely impacts on foetal and placental development, as well as progeny viability and growth after birth, highlighting this cytokine as a central maternal determinant for pregnancy outcome with clinical relevance in human fertility. Accordingly, the addition of GM-CSF to embryo culture medium elicits a significant increase in survival of transferred embryos to week 12 and live birth. Several results from different studies are consistent with a protective effect of GM-CSF on culture-induced embryo stress. GM-CSF may be particularly efficacious in women with miscarriage or early pregnancy loss history^9,10^.

Advanced IVF media containing GM-CSF cytokine have been recommended for patients with recurrent clinical and biochemical pregnancy loss, recurrent implantation failure and unexplained infertility. In this study, a retrospective cohort study was analyzed to explore the effect of GM-CSF supplemented in the routine culture medium showing a beneficial effect on embryo developmental potential from vetrificated oocytes in heterologous cycle and also on pregnancy outcome of infertile woman. We hope these results would provide new insights for improving the quality of the existing IVF culture media.

## Results

This retrospective analysis involved 25 couple with for woman factors of infertility and they were introduced in female heterologous protocol. The detailed composition of the patient’s group is shown in Table 1. The inclusion criteria for this study were the use of vitrified oocytes from donation and endometrial thickness ≥8 mm at the time of the day of oocyte thawing. The exclusion criteria were: history of chromosomal abnormalities of the men partners and cases with no oocytes survived after thawing Kitazato procedure, at last the age of patients.

**Table 1:** Clinical data of the patients

	Group I	Group II
Age of mother (years)	46.4	44.7
Pregnancy rate	40%	53%
Rate of survived oocytes	75.5%	76.8%
Rate of fertilization	66%	80%
Rate clinical pregnancy	40%	60%

The groups I and II were not statistically different for any of the parameters investigated, unless otherwise stated in the main text.

**Fig. 1:**
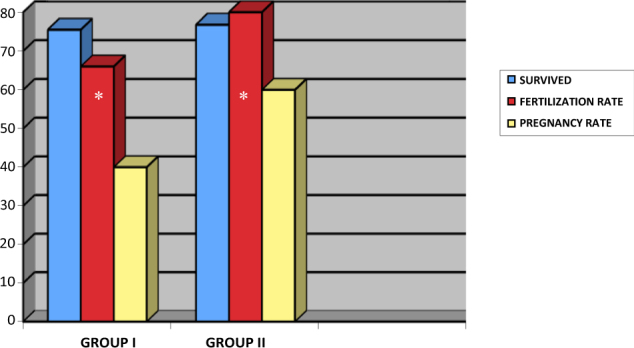
Effect of GM-CSF on  survived,  fertilization rate and  pregnancy rate. The rate of survived, fertilization and pregnancy were increased in second group of patients. For fertilization rate the differences were statistically significant as indicated (*)

The rate of survived oocytes was 75.5% in group I and 76,8% in group II. The rate of fertilization was 66% in group I and 80% in group II. Group II had the higher rate of fertilization and this difference between these rates was statistically significant (*p* ≤ 0.05), see Table 1. The results of clinical pregnancy suggested one lower rate in group I of 40% that increased in group II of the patients with 60% of positive outcomes when it has been used for oocyte and embryo cultures advanced medium. We therefore observed that the rate of fertilization and clinical pregnancy was significantly increased when the oocytes culture medium was advanced medium. The embryo quality was compared between the two groups, but there was no significant difference in cleavage rate and blastocyst formation rate. The major cases of clinical pregnancies were associated with GM-CSF culture medium but not significant correlation was observed between that two groups patients. These data reveal one higher rate in all outcomes from group II, and the associations among fertilization rate and pregnancy rate with using GM-CSF medium (Fig. 1).

## Conclusion

Advancement in vitro culture media has improved the efficacy of assisted reproductive technologies in the treatment of infertility. Culture conditions are important for the outcome of assisted reproduction technologies, as well as for the impact on pre-and post-implantation development of the human embryo. To optimize the culture conditions clinical embryologist have long been involved in a supplementation of some embryokines to the culture medium^9^. Most of these results are from animal studies^11,12^ and the effect of GM-CSF on human preimplantation embryo; these factors, secreted from epithelial cells lining the female reproductive tract, influence the development of preimplantation mouse embryos. The GM-CSF receptor mRNA was present in the fertilized oocyte and all subsequent stage of development. The clinical perinatal outcome and offspring health has yet to be elucidated^13^; nonetheless, GM-CSF deficiency in pregnancy adversely impacts fetal and placental development.

Common media among the various sequential media available for clinical use include low levels of glucose and inclusion of one or more non-essenzial amino acid, exspecially in media designed for cleavage stages. Our data suggest to include macromolecules, such as GM-CSF cytokine, in cultured media used for thawed oocytes. GM-CSF seems to show a synergistic benefit in vitro and an ability to increase the number of available and quality embryos. Finally, it is important to consider media as a part of the culture system. An inappropriate culture medium, used at wrong time of developing, can lead to impaired embryo development and even recurrent implantation failure. These results seems to contribute to indicating an improved quality of the existing culture medium and its routine application in clinical practice for infertility treatment. A larger cohort of patents would be beneficial to further support our conclusion on fertilization rate and on embryo implantation response for generating pregnancy, as well as investigate additional potential benefit on embryo quality.

## Materials and methods

We compared the laboratory and clinical outcome of 10 couples with thawed oocytes cultured with conventional media and 15 couples with thawed oocytes cultured with advanced IVF media. The vitrified oocytes came from Spanish IVF Center and the mean age of the woman for donation was 24 ± 2 years. The median number of eggs sanded for each patient was 6. The vitrification/warming protocol was performed according to the Kitazato procedure^11,14^.

Before July 2015, our routine strategy for vitrificated oocytes was to incubate in cleavage media after the ICSI procedure until the day 3 for transfer. Semen sample was performed immediately after oocyte thawing protocol. Optimization was achieved by swimming-up technique; the optimized sperms were all from the fresh ejaculated semen samples. After this time the suitable oocytes were cultured in advanced media until the intrauterine transfer. The frequency of normal fertilization was determined as the proportion of oocytes with second polar body and a pair of pronuclei and the total number of oocytes evaluated. The percentage of presumptive zygotes that cleaved (at or beyond the two-cell stage) and development to blastocysts was assessed under microscope at 48 and 125–130 h post-insemination, respectively. During the first 3 days of culture, we used medium for embryo developing, for extending cultured, we changed for all cases the cleavage medium with blastocyst culture medium. For the 10 couples performed before July 2015, we used specifically Cleavage medium (Origio) for the embryo developing until third day and then Blastassist medium (Origio) during day fourth and fifth until the transfer to blastocyst stage. This group of patients was conventionally referred to as group I. From July 2015, we change all medium with an advanced medium: Embryogen culture medium (origio) until second day, and Blastgene culture medium (Origio) until the transfer for the blastocyst developing. All oocytes of these patients, who have been cultured in advanced medium were collected and referred to as group II.

Embryo assessment did not interfere with each other and high-grade embryos were selected for transfer. Embryo transfer (ET) was normally conducted on day 3; if the number of embryos suitable for transfer was less than that of allowable for transfer (<3), transfer was conducted on day 2. There were also some patients who had more than three embryos on day 3 and it has been decided to extend the in vitro culture at day 5 or 6 at blastocyst stage.
